# Case report: An exceptional responder of low-dose continuous 5-FU in a patient with *de-novo* stage IV triple-negative breast cancer with liver and bone marrow failure

**DOI:** 10.3389/fonc.2023.1305584

**Published:** 2024-01-15

**Authors:** Bryan Chan, Jin Sun Lee, Yuan Yuan

**Affiliations:** ^1^ Internal Medicine Department, Huntington Hospital, Pasadena, CA, United States; ^2^ Department of Medicine, Cedars-Sinai Medical Center, Los Angeles, CA, United States

**Keywords:** 5-FU, stage IV breast cancer, triple negative, bone marrow failure, exceptional responder, liver failure

## Abstract

Continuous low-dose 5-FU was popularized as a therapy for pretreated metastatic breast cancer for the past few decades, spurred by the advent of the electronic infusion pump. Capecitabine, otherwise known by its trade name Xeloda, is a prodrug of 5-fluorouracil (5-FU), which is administered orally in many chemotherapy regimens, and plays a role in metastatic breast cancer treatment refractory to traditional anthracyclines and taxane therapy. In this case presentation, we describe a unique case of refractory *de-novo* stage IV triple-negative breast cancer presented with right breast primary invasive ductal carcinoma, extensive lymphadenopathy, with biopsy proven bone marrow infiltration, diffuse hepatomegaly, splenomegaly, significant hyperbilirubinemia, and bone marrow failure treated with continuous 5-FU infusion and subsequently oral capecitabine after initial treatment failure with nab-paclitaxel and sacituzimab govitecan. With this case presentation, the authors aim to showcase the versatility of 5-FU and its prodrug in treatment of metastatic triple-negative breast cancer with severe bone marrow and liver involvement while highlighting key physiologic and pharmacologic mechanisms.

## 1 Introduction

Triple-negative breast cancer (TNBC) accounts for about 15% of all newly diagnosed breast cancers worldwide and is among the most aggressive histologic subtype, with fewer treatment options and a poorer prognosis overall compared to other forms of invasive breast cancers. Demographically, TNBC tends to be more common in women younger than 40 years of age, have a BRCA mutation, or are ethnically Black. Around 6% of metastatic breast cancers arise *de novo* although, ironically, this statistic is likely lower in TNBC compared to those with HER2-positive disease (1).

As few as 20% of breast cancer patients and 40% of metastatic breast cancer patients will develop hepatic metastases at some time during their disease course. Metastatic spread to the liver often presents clearly as a well-defined mass easily diagnosed on radiographic imaging. The clinical course of these patients, particularly in those with cancer infiltration to the hepatic sinusoids, are often complicated by significant elevations in bilirubin and hypoalbuminemia and can even lead to ascites, encephalopathy, and fulminant hepatic failure. One recent case study features a patient with stage IV ER-positive breast cancer with biopsy-proven diffuse intrasinusoidal hepatic metastasis that presented with right upper quadrant abdominal pain and bilateral lower extremity swelling—weekly treatment with low-dose Adriamycin resulted in prompt reversal of her liver function testing to baseline (2).

Meanwhile, symptomatic bone marrow carcinomatosis (characterized by diffuse infiltrative growth of tumor cells in the bone marrow) is an extremely rare occurrence in patients with breast cancer, representing as few as 0.17% of cases (3). Primary clinical signs of bone marrow carcinomatosis are anemia and thrombocytopenia, often so severe as to require supplemental transfusions and bone marrow stimulants. Thus, in the event of multiorgan disease, treatment can be conceivably complex and multifaceted and may require interdisciplinary collaboration between oncologists, other sub-specialists, and hospitalist teams.

Continuous low dose 5-FU was popularized as a therapy for pretreated metastatic breast cancer for the past few decades, spurred by the advent of the medtronic infusion pump. However, there has been a dearth of recent data regarding its application among metastatic TNBC patients. In this case presentation, the authors aim to showcase the versatility of continuous 5-FU in treatment of metastatic triple negative breast cancer with severe bone marrow and liver involvement.

## 2 Case presentation

**Figure 1:**
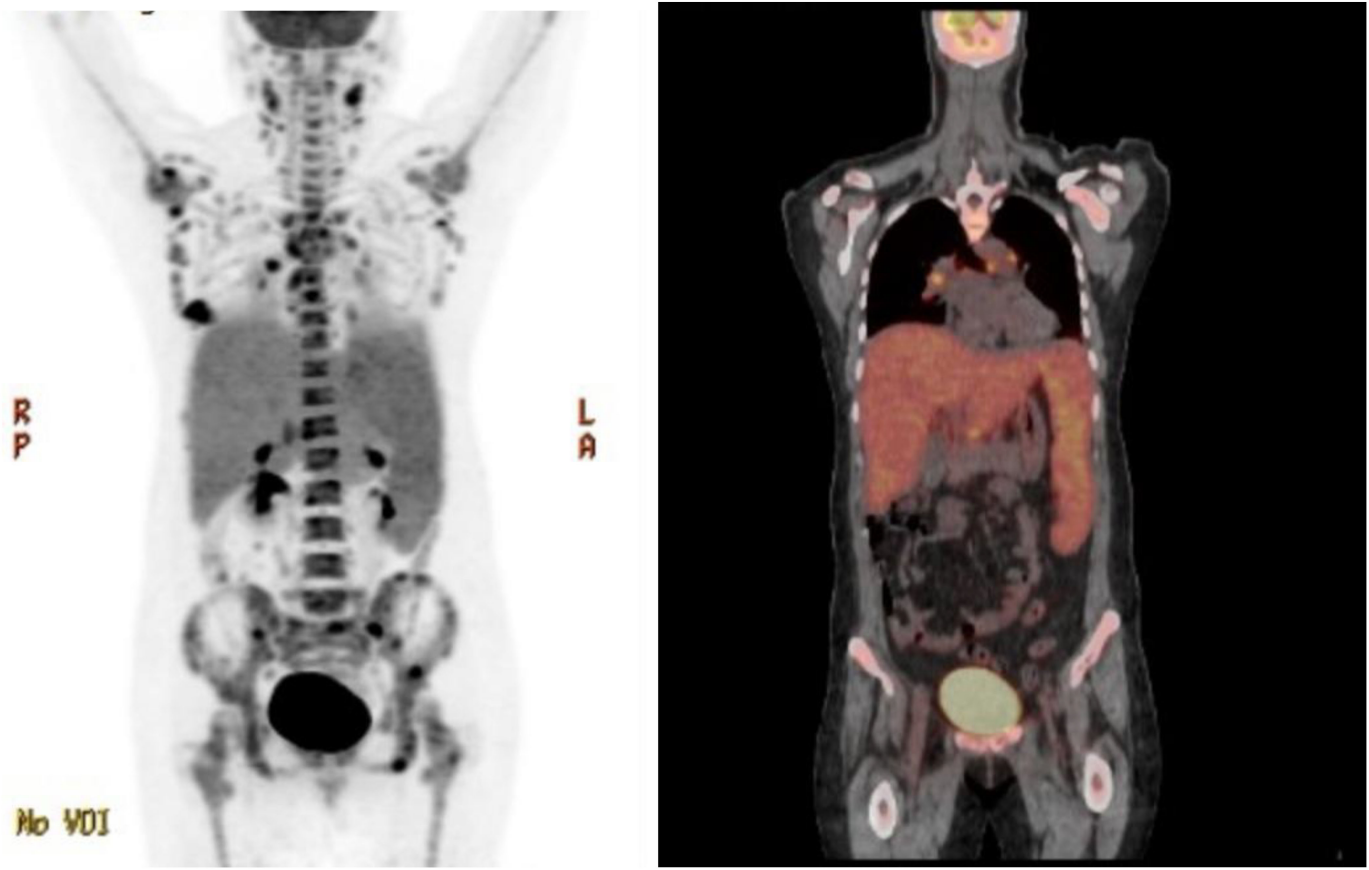
PET-CT highlighting metastatic TNBC with spread to the neck, sternum, thoracic spine, upper abdomen, and pelvis.

Patient is a 35 year-old Asian, otherwise healthy female without significant family history of cancer, who was originally diagnosed with *de-novo* stage IV triple-negative invasive ductal carcinoma via ultrasound fine needle aspiration of the right breast mass on 13/12/2022. The initial breast biopsy performed at outside clinic showed grade 3, ER negative, PR negative, HER2 IHC 2+, and negative by FISH. Subsequent fine needle aspiration of the right axillary lymph node was consistent with metastatic carcinoma of the breast on 17/12/2022. Patient was noted to have significantly decreased platelet count to 25K on 17/01/2023. A PET/CT dated 31/01/2023 showed a right hypermetabolic breast mass of 2.5 cm × 1.9 cm with extensive adenopathy in the neck, chest, pelvic, and upper abdomen (**Figure 1**). The patient was initially admitted on 20/02/2023 for evaluation of persisting vaginal bleeding and thrombocytopenia, status post-uterine artery ablation. Inpatient workup included a bone marrow biopsy, which showed metastatic breast cancer in at least 50% of the bone marrow. There were not enough biopsied tissues for PD-L1 expression test. While awaiting further tests, including PD-L1 expression, from the second biopsy, the patient started cycle 1 day 1 of weekly nab-paclitaxel and received a total of three doses dated 25/02/23, 04/03/2023, and 11/03/2023 with noted significant reduction of right breast mass but resulting pancytopenia responding to romiplostim, epoetin, and filgrastim as well as intermittent red blood cell transfusion and daily platelet transfusion. A repeat axillary lymph node biopsy confirmed high-grade metastatic carcinoma, ER 15%, PR 0%, HER2 IHC 0, Ki-67 85%. Tissue NGS did not show any targetable alteration. Genetic testing via Ambry Genetics was positive for VUS in TSC2 but otherwise negative for any other pathogenic variant. Patient finished cycle 1 of nab-paclitaxel with excellent clinical response, and the right breast mass was decreased in size to approximately 3 cm × 2 cm on palpation; the plan was to continue therapy.

**Figure 2:**
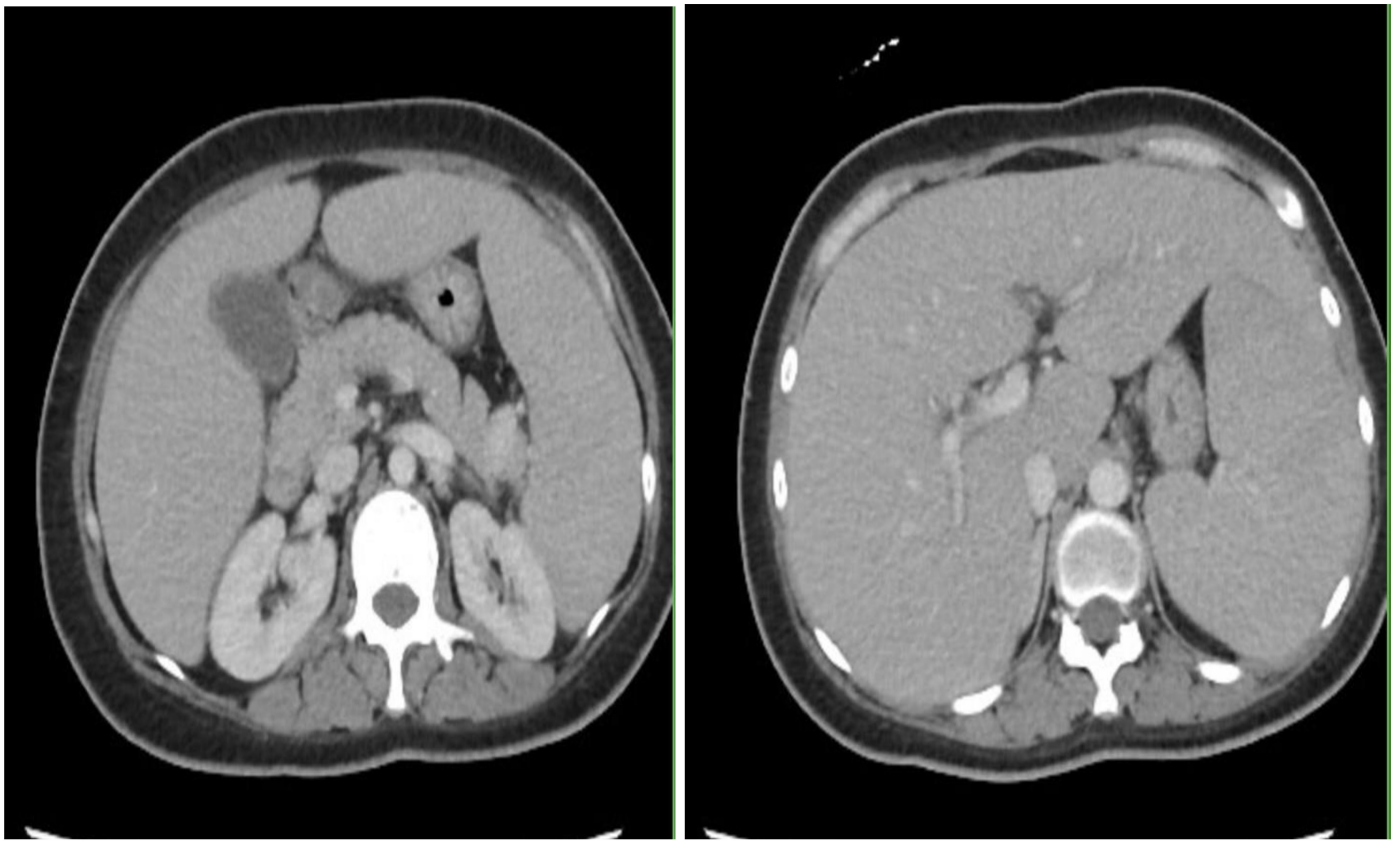
CT scan with contrast showing marked hepatosplenomegaly.

**Figure 3:**
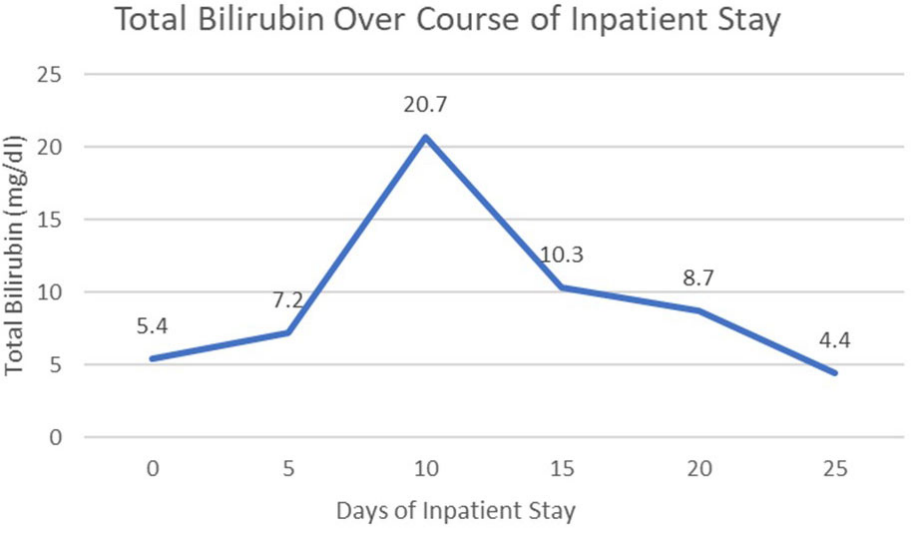
Total bilirubin over course of inpatient stay.

**Figure 4:**
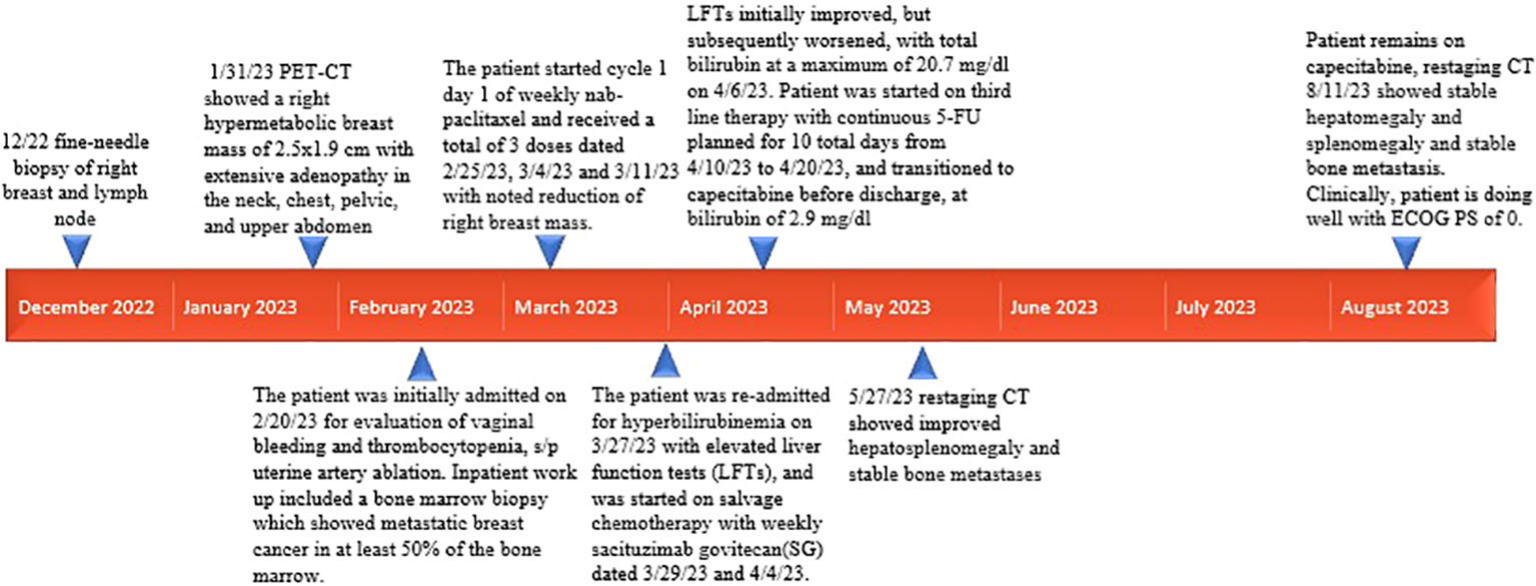
Timeline of relevant clinical events (2022–2023).

However, patient was re-admitted for hyperbilirubinemia on 27/03/2023 with a total bilirubin of 5.4 mg/dl with elevated liver function tests (AST 197 U/L/ALT 79 U/L). Patient’s vitals were collected in the ER, which showed that she was afebrile and hemodynamically stable with normal respirations and heart rate. Notable findings on exam included scleral icterus, diffuse jaundice, and a 3-cm nodular, tender breast lesion in the lateral 9 o’clock region. There was no asterixis, changes in mental status or other notable findings on the physical exam. CT scan showed diffuse hepatomegaly measuring 21.8 cm and diffuse splenomegaly, measuring 24 cm (**Figure 2**) without discrete lesions, which caused abdominal pain and bloating. Further workup failed to identify biliary ductal obstruction. Patient was subsequently started on salvage chemotherapy with weekly sacituzimab govitecan (SG) dated 29/03/2023 and 04/04/2023. Liver function testing initially improved, but subsequently worsened, with total bilirubin at a maximum of 20.7 mg/dl dated 06/04/2023. Patient was started on third-line therapy with continuous 5-FU planned for 10 total days from 10/04/2023 to 20/04/2023. The patient experienced remarkable clinical response, with total bilirubin dropping steadily and liver function testing improving daily (**Figure 3**). The patient also intermittently received inpatient radiation therapy to the liver (which resulted in transient elevations in liver function tests), requiring momentary stopping of continuous 5-FU. She was then transitioned from 5-FU to oral capecitabine after dated 21/04/2023 at a dose of 1000 mg/m^2^, 14 days on and 7 days off. Upon discharge, the patient remained on a regimen of oral capecitabine, and total bilirubin had dropped remarkably to 2.9 mg/dl. Upon follow-up at 5 months, the patient remains on capecitabine with sustained clinical response with no significant adverse side effects. 27/05/2023 restaging CT chest/abdomen/pelvis showed improve hepatomegaly at 18.4 cm, splenomegaly at 17.5 cm and stable bone metastasis. Patient was continued on capecitabine, restaging CT 11/08/2023 showed stable hepatomegaly and splenomegaly and stable bone metastasis. Clinically patient is doing well with ECOG PS of 0, lab work showed platelet count of 128k/UL, total bilirubin 1.2 mg/dl, AST 62 U/L, ALT 69 U/L without additional growth factor support (**Figure 4**).

## 3 Discussion

Here, we described a unique case of refractory *de-novo* stage IV triple-negative breast cancer presented with right breast primary invasive ductal carcinoma, extensive lymphadenopathy, with biopsy proven bone marrow infiltration, diffuse hepatomegaly, splenomegaly, significant hyperbilirubinemia, and bone marrow failure treated with continuous 5-FU infusion and subsequently oral capecitabine after initial treatment failure with nab-paclitaxel, and SG.

Bone marrow failure is a rare event in breast cancer, with symptomatic bone marrow carcinomatosis being a difficult condition to treat given the limited literature (3). The patient’s pancytopenia was of concern during the aforementioned initial hospitalization, and the patient ultimately required growth factors support with romiplostim, epoetin, and filgrastim as well as intermittent red blood cell transfusion and daily platelet transfusion. The patient’s initial treatment of a cytotoxic agent (nab-paclitaxel) likely exacerbated the patient’s precarious hematologic state, although cytopenia was present even prior to chemotherapy initiation and likely secondary to infiltration of the neoplasm to the bone marrow (resulting in bone marrow failure). In terms of histology, invasive lobular carcinomas are more likely than invasive ductal carcinomas to present with symptomatic bone marrow infiltration (4).

In addition to bone marrow carcinomatosis, the patient’s breast cancer also presented with metastatic spread to the liver with marked hepatosplenomegaly, likely a reflection of diffuse, massive intrasinusoidal infiltration of the hepatic system. Although the patient did show signs of hepatocellular damage with significant clinical markers such as significant hyperbilirubinemia, transaminitis and elevated alkaline phosphatase, the patient did not technically meet the definition of acute fulminant hepatic failure, given an INR generally below 1.5, and absence of hepatic encephalopathy on exam. Regardless, the patient’s trend of bilirubin was a useful clinical marker of treatment response in this rare presentation, given a difference of nearly 18 mg/dl before and after finishing inpatient treatment with continuous 5-FU.

It is extremely unusual that the patient had remarkable response to 5-FU and capecitabine after initial failure of salvage SG showed in this case. For patients with stage IV triple-negative right breast cancer (TNBC) without PD-L1 expression, typical first-line therapy typically consists of chemotherapy rather than chemoimmunotherapy, and both platinum and taxanes are considered appropriate options with similar outcomes (5). Second-line treatment is less standardized, but SG has emerged as a primary contender after treatment failure after first-line or second-line therapy with phase III ASCENT trial showed both progression free survival (PFS) and overall survival (OS) benefit, which led to FDA approval of the first antibody-drug conjugate in metastatic TNBC (6, 7).

Metronomic chemotherapy regimens involve continuous administration of low-dose chemotherapy agent with no or short-regular treatment-free intervals has known to offer important advantages including continuous drug exposure and significantly reduced toxicity (8, 9). The pharmacokinetic characteristics and low-toxicity profile make low-dose 5FU or capecitabine an ideal drug for metronomic administration.

Meanwhile, while continuous 5-FU has been utilized in the past for meaningful palliative results of pretreated, resistant metastatic breast cancer that failed standard first-line therapy, response rates were typically modest (10). In this case, continuous 5-FU was able to successfully “unpack” the bone marrow without increasing episodes of bleeding or an increased need for inpatient transfusions, demonstrating excellent clinical response. The mechanism of action of 5-FU is that of a thymidylate synthase (TS) inhibitor, interrupting DNA replication and thus causing oxidative stress, with rapidly dividing cells (such as cancer cells or newer cells in the bone marrow) undergoing cell death via “thymineless death” (11). This concept has also been highlighted by Banys-Paluchowski et al. in a recent review, which showed that metronomic chemotherapy may be an effective alternative to conventional chemotherapy by using low doses of continuous chemotherapy, reducing the risk of cancer cell resistance and possible disease progression (12).

Capecitabine acts as an antimetabolite that gets broken down to fluorouracil, also known as 5-FU, which ultimately interferes with the production of DNA by blocking the action of thymidylate synthase. Capecitabine has been widely adopted as an adjuvant treatment of TNBC with residual disease after neoadjuvant chemotherapy after the CREATE-X trial demonstrated disease free survival and overall survival benefits (13). The routine use of adjuvant capecitabine in early stage TNBC may have changed the potential utility of its use in metastatic or refractory setting. In addition, toxicity may often affect gastrointestinal, hematologic, and integumentary systems, and patients may require either dose-reduction or treatment discontinuation for symptom resolution; hand is a rather common adverse reaction, often involving focal irritation, erythema and peeling of the hands or feet, and is also present as an adverse reaction to various other forms of chemotherapy. The patient in this case was able to tolerate capecitabine and 5-FU without significant adverse effects such as hand–foot syndrome; biology may play a role, with patients with certain ethnic background being able to tolerate the medications better than others (14).

## 4 Conclusion

In this case presentation, we present a unique case of refractory *de-novo* stage IV TNBC with bone marrow carcinomatosis, severe thrombocytopenia, profound hyperbilirubinemia (total bilirubin over 20 mg/dl), and liver and spleen infiltration. Patient had a remarkable response to continuous metronomic dose of daily 5-FU infusion and subsequently oral capecitabine after refractory response to nab-paclitaxel and subsequent SG. Through highlighting the remarkable clinical response of the patient despite worsening metastatic disease with multiorgan sequelae, the authors hope to showcase not only the complex physiology of late stage TNBC but also better highlight the utility of metronomic 5-FU infusion and capecitabine for those with refractory disease (15). More research is needed to further understand the mechanism of action for the metronomic dosed chemotherapy agent and how to define the optimal biological dose of such an agent to market its utility in the era of immunotherapy and antibody drug conjugates.

## Data availability statement

The original contributions presented in the study are included in the article/supplementary material. Further inquiries can be directed to the corresponding author.

## Ethics statement

Written informed consent was obtained from the individual(s) for the publication of any potentially identifiable images or data included in this article.

## Author contributions

BC: Conceptualization, Validation, Writing – original draft, Writing – review & editing. JL: Writing – review & editing. YY: Conceptualization, Supervision, Validation, Writing – review & editing.
